# Evaluation of the reliability and readability of ChatGPT-4 responses regarding hypothyroidism during pregnancy

**DOI:** 10.1038/s41598-023-50884-w

**Published:** 2024-01-02

**Authors:** C. E. Onder, G. Koc, P. Gokbulut, I. Taskaldiran, S. M. Kuskonmaz

**Affiliations:** Department of Endocrinology and Metabolic Diseases, Ankara Training and Research Hospital, Ankara, Turkey

**Keywords:** Endocrinology, Endocrine system and metabolic diseases

## Abstract

Hypothyroidism is characterized by thyroid hormone deficiency and has adverse effects on both pregnancy and fetal health. Chat Generative Pre-trained Transformer (ChatGPT) is a large language model trained with a very large database from many sources. Our study was aimed to evaluate the reliability and readability of ChatGPT-4 answers about hypothyroidism in pregnancy. A total of 19 questions were created in line with the recommendations in the latest guideline of the American Thyroid Association (ATA) on hypothyroidism in pregnancy and were asked to ChatGPT-4. The reliability and quality of the responses were scored by two independent researchers using the global quality scale (GQS) and modified DISCERN tools. The readability of ChatGPT was assessed used Flesch Reading Ease (FRE) Score, Flesch-Kincaid grade level (FKGL), Gunning Fog Index (GFI), Coleman-Liau Index (CLI), and Simple Measure of Gobbledygook (SMOG) tools. No misleading information was found in any of the answers. The mean mDISCERN score of the responses was 30.26 ± 3.14; the median GQS score was 4 (2–4). In terms of reliability, most of the answers showed moderate (78.9%) followed by good (21.1%) reliability. In the readability analysis, the median FRE was 32.20 (13.00–37.10). The years of education required to read the answers were mostly found at the university level [9 (47.3%)]. Although ChatGPT-4 has significant potential, it can be used as an auxiliary information source for counseling by creating a bridge between patients and clinicians about hypothyroidism in pregnancy. Efforts should be made to improve the reliability and readability of ChatGPT.

## Introduction

Thyroid hormones play a critical role in many vital conditions such as regulation of the body’s basal metabolic rate, growth, and neural development. Hypothyroidism is characterized by thyroid hormone deficiency, and the most common cause is Hashimoto’s thyroiditis in cases where adequate iodine intake is provided. During pregnancy, the risk of hypo- and hyperthyroidism increases regardless of medical history^1,2^. Moreover, especially overt hypothyroidism has negative effects on both pregnancy and fetal health^3,4^. Therefore, the treatment and follow-up of hypothyroidism during pregnancy are crucial. Levothyroxine (LT4) is used in the treatment indicated by maternal thyroid function and thyroid peroxidase antibody (TPOAb) level. The serum thyroid stimulating hormone levels of pregnant women treated with LT4 should be closely monitored throughout the pregnancy to determine the risk of under- or overtreatment^2,5^.

Artificial Intelligence (AI) is a field within computer science dedicated to developing intelligent machines capable of emulating human-like thinking and behavior. AI systems are engineered to acquire knowledge from their surroundings and make decisions by processing the information they gather^6^. Chat Generative Pretrained Transformer (ChatGPT), first version released by OpenAI in November 2022, is a large trained language model designed to provide information on various topics over the internet using a huge amount of text data^7^. In March 2023, the latest version, ChatGPT-4, was introduced with a new feature: image evaluation. ChatGPT is successful in both open-ended questions and multiple-choice single-answer questions in the fields of medicine^8,9^. However, in terms of cancer treatment recommendations, Chen et al.^10^ reported that ChatGPT was generally unreliable in their study. In addition, Dash et al.^11^ revealed that the use of GPT-3.5 and GPT-4 to support real-world information needs in healthcare delivery responses from the GPT were largely devoid of harm, but less than only 20% of the responses agreed with the response from an informatics consultation service. Despite its promising potential, ChatGPT still has deficiencies in accuracy and knowledge.

The number of studies evaluating ChatGPT about endocrinological diseases is limited, and to the best of our knowledge, none on hypothyroidism during pregnancy^12^. Thus, this study was aimed to evaluate the reliability and readability of ChatGPT-4 answers about hypothyroidism during pregnancy using open-ended questions and patient scenarios.

## Materials and methods

### Question source and processing

In line with the recommendations on hypothyroidism and iodine in the latest guideline of the American Thyroid Association (ATA) on the diagnosis and treatment of thyroid disease during pregnancy and postpartum, a total of 19 questions were created in English by two specialists in endocrinology^13^. Disagreements between authors were adjudicated by a third specialists in endocrinology. The content of the questions consists of the negative pregnancy outcomes, treatment, follow-up strategy and treatment goals of overt and subclinical hypothyroidism. The questions were designed as open-ended questions and patient scenarios, as if formulated by patients. To remove ambiguity, all questions were grammatically edited by the two authors before being asked to ChatGPT. An example question and the answer given by ChatGPT-4 are shown in Table 1.

**Table 1:** Example question and response from ChatGPT-4.

**Question:** ‘’I am 23 years old and 5 weeks pregnant. In my thyroid function tests, TSH level was 5.4 mIU/L and my free T4 level was within the normal reference range. My thyroid antibody is positive. Should I take levothyroxine?’’
**ChatGPT-4 response:** ‘’The decision to treat subclinical hypothyroidism (characterized by a high thyroid-stimulating hormone [TSH] level but normal free thyroxine [FT4] levels) with levothyroxine during pregnancy is often made on a case-by-case basis. Your TSH level of 5.4 mIU/L is higher than the upper limit of the typical recommended range for the first trimester (which is generally about 2.5 mIU/L according to the American Thyroid Association), and you also have positive thyroid antibodies, which is associated with an increased risk of pregnancy complications. Some clinical guidelines suggest considering treatment with levothyroxine in this situation, as it may help reduce the risk of complications. However, this is a decision that needs to be made in discussion with your healthcare provider, taking into account your individual circumstances and the potential risks and benefits of treatment. In addition, even if you do not start treatment, it’s important to have your thyroid function closely monitored throughout your pregnancy. Thyroid requirements can change over the course of pregnancy, and it’s possible that you might need treatment later on. Please make sure to discuss this with your healthcare provider, who can provide the most accurate advice based on your individual situation. The information provided here should be used as a guide and does not replace professional medical advice.’’

### ChatGPT

GPT is a large language model (LLM) that generates text following a given input prompt. ChatGPT is a version of GPT-3.5 (later GPT-4) that was fine-tuned and optimized for conversations^14^. The database contains information up to the year 2021, and unlike search engines, ChatGPT generates text word by word^15^. In this study, the laterst version, ChatGPT-4, was used to answer the questions because it was reported to outperform the previous version^16^. Each question was tested twice on different days for variation in answers. In addition, a new chat session was opened in ChatGPT for each question to prevent retention bias. Reproducibility of responses was determined by categorizing responses into two categories based on the grading of each response. ChatGPT responses were categorized according to the presence or absence of misinformation. The first category included scores of “comprehensive” and “correct but inadequate” while the second category comprised scores of “some correct and some incorrect” and “completely incorrect”. A question whose responses differed in terms of accuracy and therefore were in different categories was defined as non-repeatable. This method, which evaluates the reproducibility of responses, has been used in similar studies before^17^. All questions and ChatGPT-4 answers are available online in “Supplementary materials”.

### Evaluation of reliability, quality, and readability

**Figure 1:**
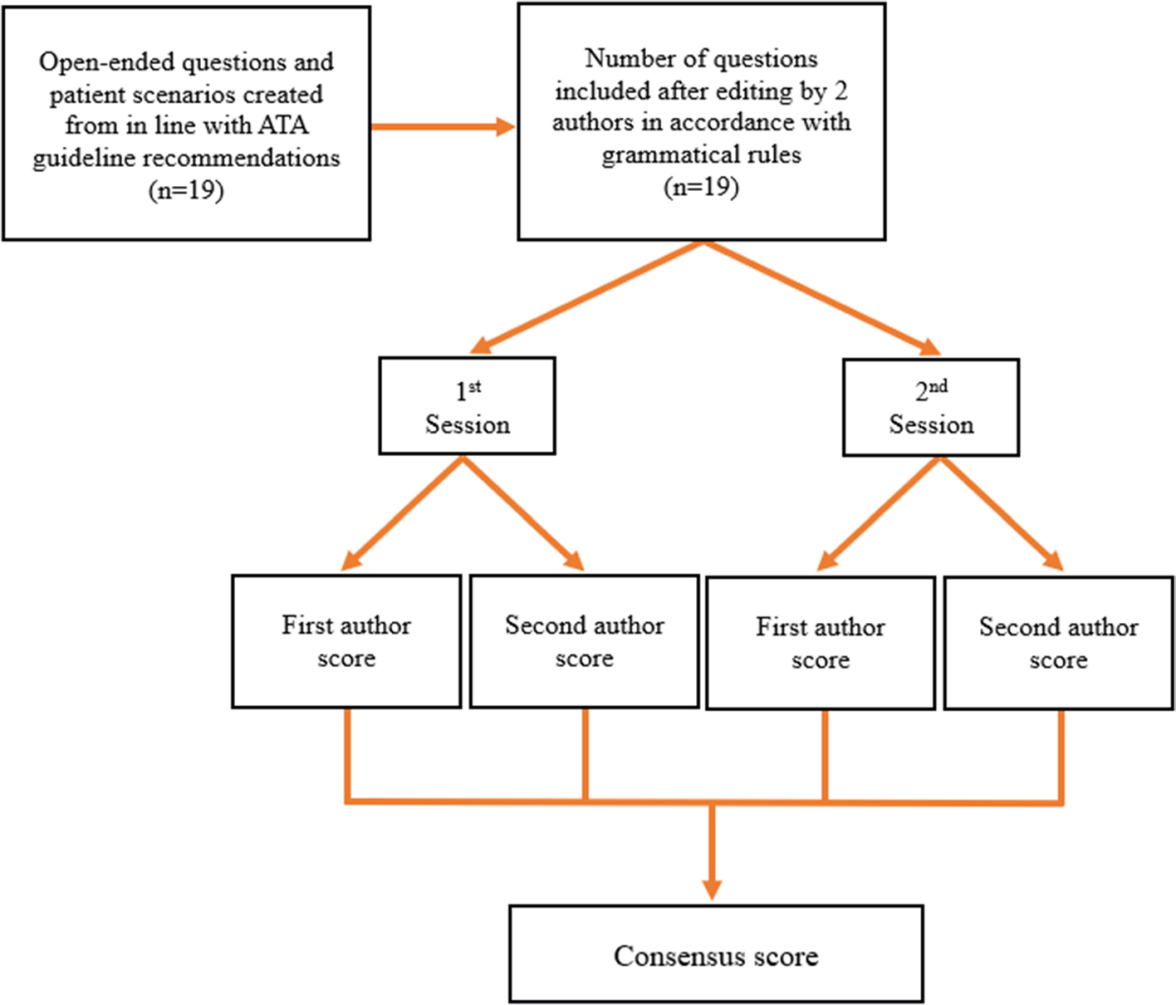
Flow chart of question selection and consensus score.

Each ChatGPT-4 response was independently evaluated by two endocrinologists with at least 10 years of experience based on ATA guidelines and clinical practice. Responses were considered misleading if they contained at least one misleading statement. Scores for reliability and quality were set by both authors. Finally, the consensus score was determined (Fig. 1).

The DISCERN scale is a three-part scale used to evaluate the reliability and quality of online health information used in previous studies^18,19^. The first section consists of eight questions to evaluate the reliability of the publication. The second section consists of seven questions to assess the quality of the information about treatment options. The last section focuses on the general quality of the publication as a source of information about treatment options. However, in this study, to evaluate the reliability of ChatGPT responses, we modified the DISCERN scale since not all of our questions were related to treatment. The modified DISCERN (mDISCERN) scale includes only the first part of the DISCERN scale (Table 2). For each question in the mDISCERN scale, the total score was calculated by scoring the no answer as 1, the partial answer as 2–3–4, and the yes answer as 5. A score below 40% (8–15) was graded as poor, 40–79% (16–31) as fair, and above 80% (32–40) as good^19^.

**Table 2:** Contents of mDISCERN, GQS and Readability indexes.

mDISCERN criteria	Total score (8–40 points)
1. Are the aims clear?	1–5 point
2. Does it achieve its aims?	1–5 point
3. Is it relevant?	1–5 point
4. Is it clear what sources of information were used to compile the publication (other than the author or producer)?	1–5 point
5. Is it clear when the information used or reported in the publication was produced?	1–5 point
6. Is it balanced and unbiased?	1–5 point
7. Does it provide details of additional sources of support and information?	1–5 point
8. Does it refer to areas of uncertanity?	1–5 point

GQS	Score
Poor quality, poor flow of the site, most information missing, not at all useful for patients	1
Generally poor quality and poor flow, some information listed but many important topics missing, of very limited use to patients	2
Moderate quality, suboptimal flow, some important information is adequately discussed but others poorly discussed, somewhat useful for patients	3
Good quality and generally good flow, most of the relevant information is listed, but some topics not covered, useful for patients	4
Excellent quality and excellent flow, very useful for patients	5

Readability indexes	Formula
Flesch reading ease score (FRE)	206.835−1015 × (W/S)−84.6 × (B/W)
Flesch–Kincaid grade level (FKGL)	0.39 × (W/S) + 11.8 × (B/W)−15.59
Simple measure of Gobbledygook (SMOG)	1.0430 × √C + 3.1291
Gunning FOG Index (GFI)	0.4 × [(W/S) + 100 × (C*/W)]
Coleman–Liau Index (CLI)	(0.0588 × L)−(0.296 × S*)−15.8

mDISCERN: Modified DISCERN, *GQS* Global quality score, *B* Number of syllables, *W* Number of words, *S* Number of sentences, *C* Complex words (≥ 3 syllables), C*: Complex words with exceptions including, proper nouns, words made three syllables by addition of ‘‘ed’’ or ‘‘es’’, compound words made of simpler words, L: Average number of letters per 100 words, S*: Average number of sentences per 100 words. When evaluating the mDISCERN criteria; 1 for no; 2,3 or 4 for partially; a score of 5 is given for yes. Partially (2–4) is preferred if the publication under review is considered to meet the criteria in question to some extent. How high or low it is rated “partially” will depend on the decision as to the extent of these deficiencies.

The previously used Global Quality scale (GQS) was applied to assess the quality of ChatGPT responses^18^. Accordingly, 1 point indicates poor quality, and 5 points indicate excellent quality (Table 2). In addition, this scale is also used for quality classification: 1–2 points representing low quality, 3 points moderate quality, and 4–5 points high quality^20^.

Finally, the readability of ChatGPT was evaluated using widely used Flesch Reading Ease (FRE) Score, Flesch–Kincaid grade level (FKGL), Gunning Fog Index (GFI), Coleman–Liau Index (CLI), and Simple Measure of Gobbledygook (SMOG) tools (Table 2)^21^. The FRE score ranges from 0 to 100; the higher the score, the easier to read the passage. FKGL is equivalent to the level of education in the United States. GFI is used to estimate the level of education required to understand the text. CLI is equivalent to the US reading grade level. Contrarily, SMOG is used to calculate the years of education an average person needs to understand any piece of writing. The FRE score drops when the readability scores increase based on FKGL, CLI, SMOG, and GFI. The FRE score accepted by the general public for texts targeting the reader is a minimum of 60, while the other 4 readability tools have a score of < 7. A score of 60–70 in the FRE corresponds to a grade level of 8–9 (age approximately 13–15 years). The five specified readability scores were calculated by copying ChatGPT’s responses for each question to the free online readability calculator tool^22^.

### Ethical approval

Since ChatGPT-4 used in the present study is a public application, and there is no human/animal participant, ethics committee approval was not required.

### Statistical analysis

The agreement between the two authors who independently evaluated the ChatGPT responses was tested using Weighted Cohen’s Kappa coefficient. Shapiro–Wilk test was used to assess the normality of the distribution. Normally distributed data were presented as mean ± SD, and non-normally distributed data as median (minimum–maximum). Categorical variables were expressed as numbers and percentages. The relationship between the data was evaluated with Pearson correlation test for parametric variables and Spearman correlation test for non-parametric variables. All statistical analyzes were performed using SPSS version 22 (IBM SPSS Statistics for Windows, Armonk, NY: IBM Corp).

## Results

### Reliability and quality

The Weighted Cohen’s Kappa coefficient between the two authors was 0.76 and 0.87 for reliability in the first and second sessions, respectively, 0.81 and 0.79, respectively, for quality.

Responses were highly consistent between the first and second sessions, and all ended with an advice to consult a healthcare professional. No misleading information was found in any of the ChatGPT-4 responses. In terms of reproducibility, the results showed that the ChatGPT provided reproducible responses (100% score for all responses) for all questions.

The mean mDISCERN score after consensus of the answers to 19 questions was 30.26 ± 3.14; the median GQS score was 4 (2–4) (Table 3). The score distribution of ChatGPT-4 responses according to the mDISCERN scale and the quality classification are presented in Table 4. Most of the answers showed moderate (78.9%) and good (21.1%) reliability. In terms of quality, the responses were mostly high quality (84.2%), followed by medium quality (10.5%). Only one response was of low quality. In this response, there was no information about iodine deficiency in pregnancy in the patient scenario created regarding the necessity of levothyroxine treatment in a 28-week pregnant patient with isolated free t4 deficiency.

**Table 3:** Summary of DISCERN, GAS and Readability results of ChatGPT-4 responses after consensus score.

Reliability, quality and readability	n = 19
Reliability	
mDISCERN score (mean ± SD)	30.26 ± 3.14
Quality	
GQS score [median (min–max)]	4 (2–4)
Readability indexes	
FRE [median (min–max)]	32.20 (13.00–37.10)
FKGL (Mean ± SD)	13.94 ± 1.55
GFI (Mean ± SD)	16.81 ± 1.69
CLI (Mean ± SD)	14.89 ± 1.44
SMOG (Mean ± SD)	12.31 ± 1.18
Reading level	
Fairly easy to read n (%)	0 (0%)
Standart/avarage n (%)	0 (0%)
Fairly difficult to read n (%)	0 (0%)
Difficult to read n (%)	12 (63.1%)
Very difficult to read n (%)	7 (36.9%)
Readers age	
8–9 years old (fourth and fifth graders) n (%)	0 (0%)
10–11 years old (fifth and sixth graders) n (%)	0 (0%)
11–13 years old (sixth and seventh graders) n (%)	0 (0%)
12–14 years old (seventh and eighth graders) n (%)	0 (0%)
13–15 years old (eighth and ninth graders) n (%)	0 (0%)
14–15 years old (ninth and tenth graders) n (%)	1 (5.3%)
15–17 years old (tenth and eleventh graders) n (%)	1 (5.3%)
17–18 years old (twelfth graders) n (%)	1 (5.3%)
18–19 years old (college level entry) n (%)	2 (10.5)
21–22 years old (college level) n (%)	9 (47.3%)
College graduate n (%)	5 (26.3%)

*mDISCERN* Modified DISCERN, *GQS* Global quality score, *FRE* Flesch Reading Ease Score, *FKGL* Flesch-Kincaid grade level, *SMOG* Simple Measure of Gobbledygook, *GFI* Gunning FOG Index, *CLI* Coleman-Liau Index. Normally distributed data are presented as mean ± SD, and non-normally distributed data are presented as median (min–max) in the table. Categorical variables were expressed as n (%).

**Table 4:** Score distribution of ChatGPT-4 responses according to the mDISCERN scale and quality classification.

	n = 19
mDISCERN criteria	
Poor (< 40% score [ < 16])	0 (0%)
Fair (40–79% score [16–31])	15 (78.9%)
Good (≥ 80% score [32])	4 (21.1%)
Quality classification	
Low quality	1 (5.3%)
Moderate quality	2 (10.5%)
High quality	16 (84.2%)

*mDISCERN* Modified DISCERN, *GQS* Global quality score. Categorical variables are presented as n (%) in the table.

### Readability

The median FRE was 32.20 (13.00–37.10), which indicated that the text was difficult to read. To understand the answers, the required level of education was college level (9 [47.3%]), followed by college graduate (5 [26.3%]). All findings are summarized in Table 3.

### Correlation analysis

mDISCERN was moderately positively correlated with GQS. However, both mDISCERN and GQS were not correlated with other readability formulas. As expected, FRE was significantly negatively correlated with other readability formulas. The correlation analysis between the reliability, quality, and readability scores of ChatGPT-4 responses is shown in detail in Table 5.

**Table 5:** Correlation analysis between reliability, quality, and readability scores of ChatGPT-4 responses.

	mDISCERN	GQS	FRE	FKGL	GFI	CLI	SMOG
mDISCERN	N/A	*p* = 0.032*	*p* = 0.708	*p* = 0.968	*p* = 0.737	*p* = 0.517	*p* = 0.535
		r = 0.491	r = 0.092	r = − 0.010	r = 0.082	r = − 0.158	r = 0.152
GQS	*p* = 0.032*	N/A	*p* = 0.833	*p* = 0.246	*p* = 0.191	*p* = 0.389	*p* = 0.129
	r = 0.491		r = − 0.052	r = − 0.280	r = − 0.314	r = 0.209	r = − 0.361
FRE	*p* = 0.708	*p* = 0.833		*p* = 0.001*	*p* = 0.046*	*p* < 0.001*	*p* = 0.039*
	r = 0.092	r = − 0.052	N/A	r = − 0.696	r = − 0.464	r = − 0.789	r = − 0.477
FKGL	*p* = 0.968	*p* = 0.246	*p* = 0.001*	N/A	*p* < 0.001*	*p* = 0.044*	*p* < 0.001*
	r = − 0.010	r = − 0.280	r = − 0.696		r = 0.947	r = 0.467	r = 0.916
GFI	*p* = 0.737	*p* = 0.191	*p* = 0.046*	*p* < 0.001*	N/A	*p* = 0.253	*p* < 0.001*
	r = 0.082	r = -0.314	r = − 0.464	r = 0.947		r = 0.276	r = 0.971
CLI	*p* = 0.517	*p* = 0.389	*p* < 0.001*	*p* = 0.044*	*p* = 0.253	N/A	*p* = 0.480
	r = − 0.158	r = 0.209	r = − 0.789	r = 0.467	r = 0.276		r = 0.173
SMOG	*p* = 0.535	*p* = 0.129	*p* = 0.039*	*p* < 0.001*	*p* < 0.001*	*p* = 0.480	N/A
	r = 0.152	r = − 0.361	r = − 0.477	r = 0.916	r = 0.971	r = 0.173	

*mDISCERN* Modified DISCERN, *GQS* Global quality score, *FRE* Flesch Reading Ease Score, *FKGL* Flesch-Kincaid grade level, *SMOG* Simple Measure of Gobbledygook, *GFI* Gunning FOG Index, *CLI* Coleman-Liau Index, *N/A* Not applicable. The correlation between the data was evaluated with Pearson correlation test for parametric variables and Spearman correlation test for non-parametric variables. *p* < 0.05 was considered statistically significant and marked with * and bolded in the table.

## Discussion

In the present study, the reliability and readability of ChatGPT-4 responses about hypothyroidism during pregnancy were evaluated using open-ended questions and patient scenarios. Our results revealed that ChatGPT-4 responses had moderate to good reliability and high quality. In terms of readability, it mostly requires a college-level education to understand that the reading level is difficult or very difficult.

Overt maternal hypothyroidism during pregnancy is associated with an increased risk of preterm birth, low birth weight, pregnancy loss, and low IQ in the children. Subclinical hypothyroidism may lead to varying degrees of adverse pregnancy outcomes. While overt hypothyroidism during pregnancy requires LT4 treatment, it is recommended that the need for treatment in subclinical hypothyroidism be decided by individual evaluation of the patients^13^. Therefore, the management of hypothyroidism in pregnancy is of great importance in terms of treatment and follow-up. Patients often have concerns about their disease and find it difficult to obtain accurate information. The rate of using the internet as the first source of reference for accessing health-related information is higher^23^. Unlike search engines (e.g., Google), ChatGPT is an advanced AI language model that has become increasingly recently popular^15^. Therefore, the reliability and readability of the responses in ChatGPT becomes important. Aiming to evaluate the accuracy and reliability of the ChatGPT model, which is used in many fields, in medical responses in their study, Johnson et al.^24^ obtained answers from ChatGPT by creating 284 medical questions that were subjectively categorized as easy, medium, and difficult by 33 physicians from 17 specialties; they reported that ChatGPT produced mostly accurate information.

ChatGPT has a wide range of uses for patients and clinicians, ranging from answering basic fact-based questions to answering complex clinical questions. In the United States Medical Licensing Examination (USMLE), ChatGPT was found to perform at or close to passing on multiple-choice single answer questions^8^. Similarly, ChatGPT answers in multiple-choice single-answer questions for the Medical School Admission Test (MSAT) were reported to be successful^25^. Contrarily, Suchman et al.^26^ used ChatGPT-3 and ChatGPT-4 to answer the 2022 and 2021 American College of Gastroenterology self-assessment tests and found that both models could not pass the test in multiple-choice single-answer questions and therefore they do not recommend using them for medical education in gastroenterology in their current form.

Upon examining the studies in which the answers of ChatGPT were evaluated with open-ended questions for patients, we found a study that obtained medical information about blepharoplasties and reported that ChatGPT-4 generally provided fast and sound medical advice while not using excessive medical jargon^27^. On self-management and education of diabetes in the field of endocrinology, Sng et al.^28^ evaluated ChatGPT’s answers to the questions under four main headings: diet and exercise, hypoglycemia and hyperglycemia education, insulin storage, and insulin administration. They found that while ChatGPT generally provided easy and accurate answers to the questions, it could also contain some incorrect statements, such as not accepting that insulin analogs can be stored at room temperature after opening^28^. Similarly, another study evaluated patient questions about obesity surgery using ChatGPT and reported that 131 (86.8%) of 151 questions were answered comprehensively^29^. Chen et al.^10^ reported that ChatGPT responses are generally unreliable in terms of cancer treatment recommendations. The high quality and moderate-good level of reliability of ChatGPT-4 responses related to hypothyroidism during pregnancy in the present study is similar to most studies in the literature^27,29^. Unlike in the study by Sng et al.^28^, no misleading information was found in the present study possibly because we used the new version of ChatGPT (ChatGPT-4), unlike Sng et al.^28^ and Chen et al.^10^. Because ChatGPT is a trained language model that learns with human feedback, its new version will have a larger database. In contrast to the studies by Sng et al.^28^ and Cox et al.^27^, the present study concludes that ChatGPT-4’s responses are not easy to understand,indicating that patients must have a certain level of education to understand the answers.

A previous study investigated the use of ChatGPT in cases requiring a more advanced multidisciplinary approach^30^ by comparing the recommendations of the multidisciplinary tumor board and ChatGPT for primary breast cancer cases. In that previous study, the results were found to have an agreement rate of 64.2%, which may be attributed to the fact that it consisted of questions containing more complex patient scenarios or that the treatment options in the oncology department change very quickly and the database of ChatGPT is limited to information until the year 2021. Lukac et al. noted that ChatGPT mostly provides general responses, but in individual therapy, the current version does not provide specific recommendations for the treatment of patients with primary breast cancer, so it cannot replace a multidisciplinary board in the real world. Another study evaluating ChatGPT models for complex clinical questions such as multidisciplinary approach was conducted by Hirosawa et al. The investigators asked the differential diagnosis for 52 clinical vignettes in internal medicine and evaluated the accuracy of top 5 and top 10 answers given by ChatGPT. The accuracy rate of ChatGPT was reported as 80% in this study. The authors emphasized the potential utility of ChatGPT-4 as a complementary tool for physicians, despite the fact that it was derived from a limited dataset of case reports of a single event^31^.

The present study determined that the reading level of ChatGPT-4 is difficult or very difficult to read, and it mostly requires a college-level education to understand the text. Contrarily, the results of the correlation analysis suggested that readability was not related to reliability and quality. These results are similar to a study by Momenaei et al.^32^ on the readability and suitability of the responses generated by ChatGPT-4 for the surgical treatment of retinal diseases; however, the questions were prepared for clinicians, so it is acceptable for the response to be at an advanced reading level. In the present study, questions targeting both patients and clinicians were created, so the development of ChatGPT-4 should be aimed to increase readability. Alternatively, separate user interfaces for patients and clinicians can be considered.

There were some limitations of our study. First, responses were analyzed only in English, so results cannot be generalized to all languages. While ChatGPT-4 is available in many languages, studies in languages other than English are few. Second, there is a lack of standardized tools for evaluating ChatGPT-4 responses. This creates heterogeneity in the comparison of studies. Third, there is no single answer to a question, although consistency has been found between the answers in different sessions. Finally, the database contains information up to 2021. As it is not connected to the internet, it is currently unable to conduct an updated literature review.

## Conclusion

In conclusion, the present study reports that ChatGPT-4 responses about hypothyroidism during pregnancy do not contain misleading information and has moderate and good reliability. In addition, the reading level is difficult or very difficult, and it requires a college-level education to understand. The difficulty of reading ChatGPT-4 will limit its easy use by the general public. Although the answers were considered safe in our study and in most of the ChatGPT-4 studies in the literature, clinicians and patients should be aware of the limitations when using the model. Although ChatGPT-4 has significant potential, it can be used as a source of auxiliary information for counseling about hypothyroidism in pregnancy. Further studies in different languages and with more comprehensive questions are needed to better evaluate ChatGPT-4 performance. Finally, efforts should be made to improve the reliability and readability of ChatGPT and to develop domain-specific models.

### Supplementary Information

Supplementary Information 1.

## Data Availability

Data generated and/or analyzed during the current study are available from the corresponding author on request.
