# Supplementary material for: Evaluation of the reliability and readability of ChatGPT-4 responses regarding hypothyroidism during pregnancy
Source: Sci Rep. 2024 Jan 2;14:243. doi: 10.1038/s41598-023-50884-w (PMC10761760; doi:10.1038/s41598-023-50884-w)
Supplement: Supplementary file 1 — Supplementary Information 1. [file 41598_2023_50884_MOESM1_ESM.pdf]

Questions and ChatGPT-4 Answers

1) Should I take an iodine supplement during pregnancy? How much iodine per day is enough?

1- The thyroid gland uses iodine to make thyroid hormones, which are critical for the normal development of a baby's brain and nervous system during pregnancy. Therefore, it's essential for pregnant women to have enough iodine.

However, the need for iodine supplementation during pregnancy may depend on several factors such as dietary intake, the iodine content in your local soil and water, and the use of iodized salt. These factors can greatly influence how much iodine you receive from your diet.

The World Health Organization (WHO) recommends a daily iodine intake of 250 micrograms (mcg) for pregnant women. The American Thyroid Association recommends that pregnant women in the United States, where iodine deficiency is not common, supplement their diet with a daily prenatal multivitamin containing 150 mcg of iodine.

However, taking too much iodine can also be harmful. Very high levels of iodine intake can actually inhibit the production of thyroid hormones, leading to transient hypothyroidism and/or goiter in the newborn.

Therefore, you should not start taking iodine supplements without first consulting with your healthcare provider. They will be able to assess your personal needs and can provide specific advice based on your individual circumstances. Please do not rely solely on the information provided here for making decisions about your health.

2) What is the safe upper limit for daily iodine consumption in pregnant women?

2- The Institute of Medicine in the United States has set the Tolerable Upper Intake Level (UL) for iodine at 1,100 micrograms (mcg) per day for adults, including pregnant women. This is the highest daily amount of iodine intake that is likely to pose no risk of adverse health effects in almost all individuals in the general population. Consuming amounts exceeding the UL on a regular basis can lead to conditions such as thyroid dysfunction.

However, it is important to remember that individual needs and tolerances can vary. Always consult with a healthcare professional before making changes to your diet or starting any supplement regimen, especially during pregnancy.

3) I am 5 weeks pregnant. My thyroid functions were within the normal range, but my thyroid antibodies were positive. How should I be followed during pregnancy?

3- Pregnancy can have various effects on thyroid function, and positive thyroid antibodies, such as anti-thyroperoxidase (TPO) antibodies and anti-thyroglobulin antibodies, can indicate an autoimmune thyroid disease such as Hashimoto's thyroiditis. Having thyroid antibodies can potentially increase the risk of complications such as miscarriage, preterm birth, or postpartum thyroiditis.

To manage this condition and monitor your health during pregnancy, your healthcare provider will likely:

1. **Regularly check your thyroid function:** Even if your thyroid function is normal at the moment, your healthcare provider will want to monitor it closely throughout your pregnancy. This usually involves blood tests to check your levels of thyroid-stimulating hormone (TSH) and free thyroxine (FT4). The frequency of these tests may depend on various factors, including your current thyroid function, your symptoms, and whether or not you're taking thyroid hormone medication.
2. **Consider treatment if necessary:** If your thyroid function becomes abnormal, your healthcare provider may recommend treatment with thyroid hormone medication. The goal of treatment is to normalize your thyroid function, which can help reduce the risk of pregnancy complications.
3. **Monitor your symptoms:** Along with regular blood tests, your healthcare provider will also likely ask about any symptoms you may be experiencing, such as fatigue, weight changes, or depression. These symptoms can sometimes indicate a problem with your thyroid function.
4. **Postpartum follow-up:** Women with thyroid antibodies have an increased risk of postpartum thyroiditis, a condition in which the thyroid becomes inflamed after childbirth. Your healthcare provider will likely want to continue monitoring your thyroid function after you give birth.

It's important to maintain open communication with your healthcare provider and promptly report any new or worsening symptoms. Each person's situation is unique, and your healthcare provider can provide the most accurate information

based on your individual circumstances. The aforementioned steps are the general guidelines, and the actual plan may vary depending on your specific needs and health condition.

4) I found out that I was 7 weeks pregnant. My thyroid antibodies are positive but my thyroid functions are within the normal reference range. Am I at risk of miscarriage?

4- Thyroid autoimmunity, indicated by the presence of thyroid antibodies, has been associated with an increased risk of miscarriage, even when thyroid function is normal. The most common antibodies involved are anti-thyroid peroxidase (anti-TPO) and anti-thyroglobulin antibodies.

However, while the risk may be higher, it's important to understand that this doesn't mean a miscarriage will definitely happen. Many women with positive thyroid antibodies have successful pregnancies.

You should share your concerns with your healthcare provider. They might closely monitor your thyroid function and the progression of your pregnancy. They can also provide advice on potential treatment options. For example, some studies have suggested that treatment with the thyroid hormone levothyroxine may reduce the risk of miscarriage in women with thyroid autoimmunity, but this is an area of ongoing research, and the approach can vary based on individual circumstances.

It's crucial to follow your healthcare provider's advice and have regular prenatal check-ups to ensure that both you and your baby are healthy throughout your pregnancy. Remember that each person's situation is unique, and what works best will depend on a variety of factors, including your overall health, your medical history, and the specifics of your current situation.

Please note that while I strive to provide accurate and up-to-date information, you should always consult with your healthcare provider for personalized medical advice.

5) Is there an association between thyroid autoantibody positivity and premature delivery?

5- Yes, several studies suggest that there is an association between thyroid autoantibody positivity (most commonly anti-thyroid peroxidase [TPO] and anti-thyroglobulin antibodies) and an increased risk of preterm delivery, even in women who have normal thyroid function.

The mechanism of this association is not fully understood, but it's believed that thyroid autoantibodies might reflect a generalized autoimmune disorder which could affect the pregnancy.

However, it's important to remember that while the risk is increased, it doesn't mean that preterm delivery will necessarily occur. Many women with positive thyroid antibodies have full-term pregnancies.

Your healthcare provider may want to monitor your pregnancy more closely if you have positive thyroid antibodies. They can provide advice on any potential treatment options, and also help monitor for signs of preterm labor.

Keep in mind that while there is an association, each person's situation is unique, and the specifics of your individual health, lifestyle, and pregnancy can all influence your risk of preterm delivery. As always, for personalized medical advice, please consult with your healthcare provider.

6) What is the definition of hypothyroidism in pregnancy?

6- Hypothyroidism is a condition where the thyroid gland does not produce enough thyroid hormone. Thyroid hormones are critical for normal development of the baby's brain and nervous system, as well as for the mother's metabolic needs during pregnancy.

The definitions of hypothyroidism during pregnancy have evolved with our understanding of thyroid physiology in pregnancy and are a topic of ongoing research and discussion. However, hypothyroidism in pregnancy is typically classified into two types: overt and subclinical.

1. **Overt Hypothyroidism:** This is defined as elevated thyroid-stimulating hormone (TSH) levels and low free thyroxine (FT4) levels. It's a clear indication that the thyroid is not producing enough thyroid hormones.

2. **Subclinical Hypothyroidism:** This is defined as elevated TSH levels but normal FT4 levels. In this case, the thyroid is still able to produce enough thyroid hormones to keep FT4 in the normal range, but it requires more stimulation (higher TSH levels) to do so.

For non-pregnant individuals, the reference range for TSH is typically around 0.4 - 4.0 mIU/L, but these values can change during pregnancy. The American Thyroid Association (ATA) recommends a trimester-specific reference range for pregnant women, which is generally a bit lower:

- First trimester: less than 2.5 with a range of 0.1-2.5 mIU/L
- Second trimester: 0.2-3.0 mIU/L
- Third trimester: 0.3-3.0 mIU/L

These are general guidelines, and the specific reference ranges may vary depending on the laboratory and population.

If a pregnant woman is diagnosed with hypothyroidism, treatment typically involves taking a synthetic form of the thyroid hormone levothyroxine to restore the body's normal thyroid hormone levels. It's important to note that uncontrolled hypothyroidism during pregnancy can lead to various complications, such as preterm birth, low birth weight, and even loss of pregnancy. Therefore, prompt diagnosis and treatment are crucial.

Always consult with your healthcare provider for personalized medical advice. Your provider can provide the most accurate information based on your individual circumstances.

7) Should women with overt hypothyroidism be treated during pregnancy?

7- Yes, overt hypothyroidism should be treated during pregnancy. Untreated hypothyroidism, especially in its overt form, can lead to a number of complications for both the mother and baby, including preterm birth, preeclampsia, miscarriage, low birth weight, and even developmental issues in the child.

The standard treatment for hypothyroidism, including during pregnancy, is levothyroxine, a synthetic form of the thyroid hormone thyroxine (T4). The goal of treatment is to restore the mother's thyroid hormone levels to the normal range, thereby reducing the risk of complications.

Dosage needs to be carefully monitored, as both too much and too little thyroid hormone can cause problems. The dose of levothyroxine is often increased during pregnancy, due to increased demands for thyroid hormone.

Women with overt hypothyroidism who are planning to become pregnant or who are already pregnant should work closely with their healthcare provider to manage their condition. This often involves regular blood tests to monitor thyroid function and adjust the medication dosage as necessary.

As always, the specifics of any medical condition and treatment plan should be discussed with a healthcare provider who can provide personalized advice based on individual circumstances.

8) Should women with subclinical hypothyroidism be treated during pregnancy?

8- The management of subclinical hypothyroidism, defined as high thyroid-stimulating hormone (TSH) levels with normal free thyroxine (FT4) levels, during pregnancy is a topic of ongoing research and debate.

Many clinical guidelines suggest considering treatment with levothyroxine for pregnant women with subclinical hypothyroidism, particularly if they are symptomatic, if the TSH is above a certain level (often 10 mIU/L is used as a threshold), or if they have positive thyroid autoantibodies. This is due to the association of subclinical hypothyroidism with an increased risk of miscarriage, premature birth, and other adverse pregnancy outcomes, especially in the presence of thyroid autoantibodies.

However, some studies have suggested that treating subclinical hypothyroidism does not necessarily improve pregnancy outcomes, and overtreatment can also have negative effects. Therefore, the decision to treat subclinical hypothyroidism during pregnancy is often made on a case-by-case basis, taking into account factors such as the woman's symptoms, the exact TSH level, the presence of thyroid autoantibodies, and the potential risks and benefits of treatment.

It's important for women with subclinical hypothyroidism who are pregnant or planning to become pregnant to have a discussion with their healthcare provider about the best course of action. Regular monitoring of thyroid function throughout pregnancy is often recommended.

As always, individual healthcare needs should be addressed with a healthcare provider who can provide personalized advice based on individual circumstances. The information provided here should be used as a guide and does not replace professional medical advice.

9) I am 23 years old and 5 weeks pregnant. In my thyroid function tests, TSH level was 5.4 mIU/L and my free T4 level was within the normal reference range. My thyroid antibody is positive. Should I take levothyroxine?

9- The decision to treat subclinical hypothyroidism (characterized by a high thyroid-stimulating hormone [TSH] level but normal free thyroxine [FT4] levels) with levothyroxine during pregnancy is often made on a case-by-case basis.

Your TSH level of 5.4 mIU/L is higher than the upper limit of the typical recommended range for the first trimester (which is generally about 2.5 mIU/L according to the American Thyroid Association), and you also have positive thyroid antibodies, which is associated with an increased risk of pregnancy complications.

Some clinical guidelines suggest considering treatment with levothyroxine in this situation, as it may help reduce the risk of complications. However, this is a decision that needs to be made in discussion with your healthcare provider, taking into account your individual circumstances and the potential risks and benefits of treatment.

In addition, even if you do not start treatment, it's important to have your thyroid function closely monitored throughout your pregnancy. Thyroid requirements can change over the course of pregnancy, and it's possible that you might need treatment later on.

Please make sure to discuss this with your healthcare provider, who can provide the most accurate advice based on your individual situation. The information provided here should be used as a guide and does not replace professional medical advice.

10) I am 28 years old and 6 weeks pregnant. In my thyroid function tests, TSH level was 3.5 mIU/L and my free T4 level was within the normal reference range. My thyroid antibody is positive. Should I take levothyroxine?

10- The question of whether to treat subclinical hypothyroidism (characterized by a slightly high thyroid-stimulating hormone [TSH] level but normal free thyroxine [FT4] levels) with levothyroxine during pregnancy is a topic of ongoing research and debate.

Your TSH level of 3.5 mIU/L is within the reference range used for non-pregnant adults, but is slightly higher than the upper limit of the typical recommended range for the first trimester (which is generally about 2.5 mIU/L according to the American Thyroid Association). However, the precise upper limit can vary depending on different guidelines and the population studied.

You also have positive thyroid antibodies, which can be associated with an increased risk of pregnancy complications, including the risk of progressing to overt hypothyroidism.

Some guidelines suggest considering treatment with levothyroxine in situations like yours, but this is not universally agreed upon and the evidence is not entirely clear. This decision is typically made on a case-by-case basis, taking into account factors such as your symptoms, the exact TSH level, the presence of thyroid autoantibodies, and the potential risks and benefits of treatment.

You should definitely discuss this situation with your healthcare provider. They can provide the most accurate advice based on your individual circumstances. In any case, it's important to have your thyroid function closely monitored throughout your pregnancy, given your positive thyroid antibodies.

The information provided here should be used as a guide and does not replace professional medical advice. Always consult with your healthcare provider for personalized medical advice.

11) I am 26 years old and 8 weeks pregnant. In my thyroid function tests, the TSH level was 2 mIU/L and the free T4 level was within the normal reference range. My thyroid antibody is positive. Should I take levothyroxine?

11- The management of thyroid health during pregnancy can be complex, particularly in cases where thyroid antibodies are present. Thyroid autoimmunity, indicated by the presence of thyroid antibodies, is associated with an increased risk of some pregnancy complications, such as miscarriage and preterm birth, even if thyroid hormone levels are normal.

However, your thyroid-stimulating hormone (TSH) level of 2 mIU/L and your normal free thyroxine (FT4) levels are within the recommended range for pregnancy according to guidelines from organizations like the American Thyroid Association. This indicates that your thyroid is currently functioning properly.

The decision to treat with levothyroxine in your case is a subject of debate among experts. Some suggest considering levothyroxine treatment for pregnant women with normal thyroid function who are thyroid antibody positive, in an effort to potentially decrease the risk of complications. However, this is not universally agreed upon, and more research is needed to definitively show the benefits.

It's essential for you to discuss this with your healthcare provider who can take into account your full medical history, overall health, the potential risks and benefits of treatment, and the latest research. Regardless of the decision about levothyroxine, it's likely that your provider will want to closely monitor your thyroid function throughout your pregnancy given the presence of thyroid antibodies.

As always, consult with your healthcare provider for personalized medical advice. The information provided here should be used as a guide and does not replace professional medical advice.

12) I am 27 years old and 6 weeks pregnant. In my thyroid function tests, TSH level was 6 mIU/L and my free T4 level was within the normal reference range. My thyroid antibody is negative. Should I take levothyroxine?

12- In your case, with a thyroid-stimulating hormone (TSH) level of 6 mIU/L and normal free thyroxine (FT4) levels, you would be classified as having subclinical hypothyroidism. While this is within the normal range for non-pregnant individuals, it's higher than the upper limit of the typical recommended range for the first trimester of pregnancy (which is generally about 2.5 mIU/L according to the American Thyroid Association). However, the exact upper limit can vary depending on different guidelines and the population studied.

The management of subclinical hypothyroidism during pregnancy is a topic of ongoing research and debate. While some guidelines suggest treating subclinical hypothyroidism during pregnancy to possibly reduce the risk of complications, other studies have suggested that treating subclinical hypothyroidism does not necessarily improve pregnancy outcomes.

In your case, your thyroid antibodies are negative, which indicates that you are not currently showing signs of autoimmune thyroid disease. This might influence the decision on whether to start treatment, as the presence of thyroid antibodies can be associated with a higher risk of complications and might make treatment more likely.

Ultimately, the decision to treat subclinical hypothyroidism during pregnancy should be made in discussion with your healthcare provider, taking into account your individual circumstances and the potential risks and benefits of treatment.

It's important to have your thyroid function closely monitored throughout your pregnancy, given your elevated TSH level. Your healthcare provider can provide the most accurate advice based on your individual situation. The information provided here should be used as a guide and does not replace professional medical advice.

13) What adverse outcomes are associated with overt hypothyroidism during pregnancy?

13- Overt hypothyroidism, characterized by high thyroid-stimulating hormone (TSH) levels and low free thyroxine (FT4) levels, can be associated with a number of adverse outcomes if not properly managed during pregnancy. These complications can affect both the mother and the baby:

Maternal complications:

1. **Miscarriage**: Untreated or inadequately treated hypothyroidism can increase the risk of miscarriage.
2. **Preeclampsia**: Women with untreated hypothyroidism may be at higher risk for developing preeclampsia, a serious pregnancy complication characterized by high blood pressure and signs of damage to another organ system, most often the liver and kidneys.
3. **Anemia**: Hypothyroidism is often associated with anemia (low red blood cell count), which can cause fatigue and other issues.
4. **Placental abruption**: This is a serious condition in which the placenta separates from the uterus before childbirth.
5. **Postpartum hemorrhage**: Hypothyroidism may increase the risk of excessive bleeding after delivery.

Fetal and neonatal complications:

1. **Preterm birth**: Hypothyroidism in the mother can increase the risk of premature delivery.
2. **Low birth weight**: Babies born to mothers with untreated hypothyroidism may weigh less than normal.
3. **Stillbirth**: The risk of stillbirth may be increased in pregnancies complicated by hypothyroidism.
4. **Developmental issues**: Adequate thyroid hormones are essential for fetal brain development. Untreated hypothyroidism, particularly in the early stages of pregnancy, can potentially lead to lower IQ and developmental issues in the child.

It's important to note that these risks are significantly reduced when hypothyroidism is properly treated. Women with known hypothyroidism who become pregnant should work closely with their healthcare provider to ensure their thyroid hormone levels are adequately managed throughout the pregnancy. Regular monitoring and appropriate adjustment of medication (usually levothyroxine) is necessary in such cases.

The information provided here is a general guide and does not replace professional medical advice. Always consult with your healthcare provider for personalized medical advice.

14) What adverse outcomes are associated with subclinical hypothyroidism during pregnancy?

14- Subclinical hypothyroidism, characterized by mildly elevated thyroid-stimulating hormone (TSH) levels with normal free thyroxine (FT4) levels, can also be associated with several adverse outcomes during pregnancy. While the risks are generally lower than with overt hypothyroidism, untreated subclinical hypothyroidism can still have potential implications for both the mother and baby.

Maternal complications:

1. **Miscarriage**: There is some evidence suggesting that subclinical hypothyroidism may increase the risk of miscarriage, although more research is needed.
2. **Preeclampsia**: Some studies suggest an increased risk of preeclampsia in women with subclinical hypothyroidism.
3. **Placental abruption**: Although less common, there might be a slight increase in the risk of placental abruption.

Fetal and neonatal complications:

1. **Preterm birth**: Some studies have found a higher rate of preterm birth in women with subclinical hypothyroidism.
2. **Low birth weight**: There may be an increased risk of low birth weight in babies born to mothers with subclinical hypothyroidism.
3. **Impaired neuropsychological development**: While most commonly associated with overt hypothyroidism, some research suggests that even milder forms of maternal thyroid deficiency could potentially impact the child's cognitive development. This is still an area of ongoing research.

The presence of thyroid autoantibodies, which often occurs in women with subclinical hypothyroidism, can further increase the risk of these complications.

Again, it's important to note that these risks can be reduced with appropriate management. The decision to treat subclinical hypothyroidism during pregnancy is typically made on a case-by-case basis, considering factors such as the woman's TSH level, the presence of symptoms, the presence of thyroid autoantibodies, and other individual circumstances.

Regular monitoring of thyroid function throughout pregnancy is generally recommended for women with subclinical hypothyroidism.

As always, consult with your healthcare provider for personalized medical advice. The information provided here should be used as a guide and does not replace professional medical advice.

15) I am 28 weeks pregnant, laboratory tests revealed only free T4 deficiency. Should I take levothyroxine treatment?

15- If your free thyroxine (FT4) levels are low during pregnancy, this may indicate hypothyroidism, even if your thyroid-stimulating hormone (TSH) level is within normal limits. Thyroid hormones, including thyroxine (T4), are important for fetal development, particularly brain development, as well as for maintaining a healthy pregnancy.

The exact interpretation of your thyroid function tests, however, will depend on the exact levels, the reference ranges used by the lab, and any symptoms you may be experiencing. If your healthcare provider confirms that you have low FT4 levels and diagnoses you with hypothyroidism, they will most likely recommend treatment with levothyroxine.

Levothyroxine is a synthetic form of the thyroid hormone thyroxine (T4). The goal of treatment is to bring your thyroid hormone levels back into the normal range, thereby reducing the risk of complications. The dosage of levothyroxine is carefully adjusted based on regular blood tests to ensure that your thyroid hormone levels are kept within the target range.

Please make sure to discuss this with your healthcare provider, who can provide the most accurate advice based on your individual situation. The information provided here should be used as a guide and does not replace professional medical advice.

16) What is the biochemical target in the treatment of hypothyroidism in pregnant women?

16- In the treatment of hypothyroidism in pregnant women, the biochemical target is generally to maintain the thyroid-stimulating hormone (TSH) and free thyroxine (FT4) levels within the reference range suitable for each trimester of pregnancy.

The American Thyroid Association guidelines recommend the following TSH targets during pregnancy:

- First trimester: 0.1-2.5 mIU/L
- Second trimester: 0.2-3.0 mIU/L
- Third trimester: 0.3-3.0 mIU/L

The target for FT4 is usually to maintain it within the reference range provided by the lab for pregnant women.

Levothyroxine is typically used to treat hypothyroidism during pregnancy. Once therapy is initiated, the woman's TSH and FT4 levels should be monitored every 4-6 weeks, and the levothyroxine dose should be adjusted as necessary to keep these levels within the target ranges.

It's important to note that these are general targets, and the exact therapeutic goals may vary depending on the individual's clinical situation and the guidance of their healthcare provider. Always consult with your healthcare provider for personalized medical advice. The information provided here should be used as a guide and does not replace professional medical advice.

17) I have primary hypothyroidism and am on levothyroxine therapy. I just found out that I am pregnant. How often should I be followed during my pregnancy?

17- Congratulations on your pregnancy!

Pregnancy often leads to changes in the body's demand for thyroid hormones, so it's important to closely monitor your thyroid function throughout this time.

Women with pre-existing hypothyroidism, like you, often need to increase their dose of levothyroxine during pregnancy. In fact, the American Thyroid Association recommends an increase in the levothyroxine dose by approximately 20-30% as soon as pregnancy is confirmed.

Once your levothyroxine dose has been adjusted, it's typically recommended to have thyroid function tests (including thyroid-stimulating hormone [TSH] and free thyroxine [FT4]) performed every 4-6 weeks during pregnancy. Your healthcare provider may adjust your levothyroxine dose based on these tests to maintain your TSH and FT4 within the appropriate range for each trimester.

After delivery, your levothyroxine dose will usually need to be reduced back to your pre-pregnancy dose, and your thyroid function should be checked again 6 weeks postpartum.

Keep in mind that these are general recommendations. Always follow your healthcare provider's advice for monitoring and treatment adjustments, as individual needs may vary. The information provided here should be used as a guide and does not replace professional medical advice.

18) Before pregnancy, I was using levothyroxine treatment for hypothyroidism, I found out that I am 4 weeks pregnant. Should I make changes to my levothyroxine treatment?

18- Yes, adjustments to your levothyroxine dosage are often needed once you become pregnant. Pregnancy increases the body's demand for thyroid hormones, which often necessitates a higher dose of levothyroxine.

In fact, the American Thyroid Association recommends that women with hypothyroidism who become pregnant should increase their levothyroxine dose by approximately 20-30% as soon as pregnancy is confirmed. This often translates into taking two additional tablets of levothyroxine per week (if you take one daily).

However, do not make any changes to your medication regimen without first consulting your healthcare provider. You should get in touch with them as soon as possible to discuss your pregnancy and the necessary adjustments to your treatment plan. They can provide personalized advice based on your specific circumstances and current levothyroxine dose.

After adjusting your dose, your provider will likely recommend regular thyroid function tests (including thyroid-stimulating hormone [TSH] and free thyroxine [FT4]) throughout your pregnancy, typically every 4-6 weeks, to ensure your levels are in the appropriate range.

The information provided here should be used as a guide and does not replace professional medical advice. Always consult with your healthcare provider for personalized medical advice.

19) Should I have additional maternal or fetal testing during pregnancy other than monitoring my thyroid function for adverse effects for me and my baby?

19- Women with hypothyroidism, either overt or subclinical, are generally closely monitored during pregnancy, with regular thyroid function tests (TSH and FT4) performed approximately every 4-6 weeks to ensure adequate control of thyroid hormone levels.

Besides the regular thyroid function monitoring, there are no specific additional maternal or fetal tests that are universally recommended solely due to maternal hypothyroidism, as long as thyroid hormone levels are kept within the appropriate range. However, the need for additional testing would depend on various factors including your overall health, history of previous pregnancies, presence of other health conditions, and the health of your baby.

For instance, if your healthcare provider has concerns about the possibility of a high-risk pregnancy due to other reasons, such as advanced maternal age or pre-existing health conditions (like diabetes, hypertension), they might recommend

additional monitoring and testing. This could include more frequent ultrasounds, non-stress tests, or other types of prenatal screening.

Likewise, if there are concerns about fetal growth or well-being (which could be a concern if your hypothyroidism is not well-controlled), additional fetal testing may be needed.

In any case, regular prenatal care and close communication with your healthcare provider are crucial to ensure both your health and the health of your baby. You should always follow your healthcare provider's recommendations for prenatal testing and care.

Remember, the information provided here should be used as a guide and does not replace professional medical advice. Always consult with your healthcare provider for personalized medical advice.
